# Crystal structure of {μ-6,6′-dimeth­oxy-2,2′-[ethane-1,2-diylbis(nitrilo­methanylyl­idene)]diphenolato}(meth­anol)(nitrato)nickel(II)sodium

**DOI:** 10.1107/S160053681402159X

**Published:** 2014-10-08

**Authors:** Olesia V. Moroz, Viktor A. Trush, Tatiana Yu. Sliva, Irina S. Konovalova, Vladimir M. Amirkhanov

**Affiliations:** a Taras Shevchenko National University of Kyiv, Department of Chemistry, 64/13 Volodymyrska Street, Kyiv 01601, Ukraine; b STC "Institute for Single Crystals", National Academy of Science of Ukraine, 60 Lenina Avenue, Kharkiv 61001, Ukraine

**Keywords:** crystal structure, hydrogen bonds, π–π stacking, Ni^II^–Na heterometallic complex, Schiff base

## Abstract

In the molecular structure of the title compound, [NaNi(C_18_H_18_N_2_O_4_)(NO_3_)(CH_3_OH)], the Ni^2+^ ion has a slightly distorted square-planar coordination environment defined by two N and two O atoms which belong to a Schiff base ligand, *viz.* 6,6′-dimeth­oxy-2,2′-[ethane-1,2-diylbis(nitrilo­methanylyl­idene)]diphenolate. Seven O atoms form the coordination environment of the Na^+^ ion: four from the Schiff base ligand, two from a bidentate chelating nitrate anion and one O atom from a coordinating methanol mol­ecule. In the crystal, the bimetallic complexes are assembled into chains along the *b*-axis direction *via* weak C—H⋯O hydrogen-bond inter­actions. Neighbouring chains are in turn connected through bifurcated O—H⋯O hydrogen bonds that involve the coordinating methanol mol­ecules and the nitrate anions, and through π–π stacking inter­actions between phenyl rings of neighbouring mol­ecules.

## Abstract 2

Two phenolate O atoms provided by a Schiff base ligand create a double bridge between Ni^2+^ and Na^+^ ions. The coordination environment of the Ni^2+^ ion is square-planar and it has an unusual seven-coordinated geometry: four atoms from the Schiff base ligand, two from a nitrate anion, which coordinates in a bidentate chelating mode, and one O atom from the coordinated methanol mol­ecule. C—H⋯O weak hydrogen-bond inter­actions result in the formation of chains along the *b*-axis direction which are further assembled by bifurcated O—H⋯O hydrogen bonds and π-stacking inter­actions.

## Chemical context   

Schiff bases are known to be effective ligands able to coord­inate a wide range of different metal ions, and they have been widely utilized in the study of biochemical processes (Lindoy *et al.*, 1976 ▶; Correia *et al.*, 2005 ▶). Compartmental Schiff base ligands, *i.e.* tetra- and hexa­dentate Schiff base ligands with different ‘compartments’ for different types of metal ions, have been employed extensively as ‘blocking ligands’. Typical examples would be *e.g.* ligands with an N_2_O_4_ donor set with two Schiff base N-donor sites, two anionic phenolate donor sites, and two additional ether donor sites. The N_2_O_2_ compartment is generally more favorable for 3*d* metal ions. The additional O-donor atoms provide the opportunity to accommodate a second metal ion, which might be a 3*d*-, 4*f*-, *s*- or *p*-block element, thus allowing the production of di-, tri- or oligonuclear systems (Gheorghe *et al.*, 2006 ▶; Costes *et al.*, 2008 ▶; Andruh *et al.*, 2009 ▶).

Studies on heterometallic complexes began at the end of the 1960s. They are of inter­est because of their physicochemical properties that arise from the presence of dissimilar metal ions in close proximity. The majority of publications in this field are devoted to the preparation of 3*d*–4*f* heterometallic complexes (Costes *et al.*, 1998 ▶; Koner *et al.*, 2005 ▶; Sakamoto *et al.*, 2001 ▶). Metal salicylaldimines, on the other hand, represent a fascin­ating group of ligands that are not only effective complexing agents for *p*- and *d*-block elements, but also for alkali metal ions similar to the more well known ligand systems such as crown ethers, cryptands *etc*. Much of the inter­est concerning the coordination chemistry of alkali metal ions originates from the development of mol­ecular systems that can mimic natur­ally occurring mol­ecules that are responsible for the selective transport of these ions, *e.g.* through membranes. Some of the alkali–metal-ion adducts behave as precursors for other potentially inter­esting mol­ecular species that can be used for small-mol­ecule activation (Gambarotta *et al.*, 1982 ▶), electron storage (Gallo *et al.*, 1997 ▶) and the production of materials with remarkable magnetic properties, the alkali cation being crucial in determining the three-dimensional network in the solid state (Miyasaka *et al.*, 1996 ▶).
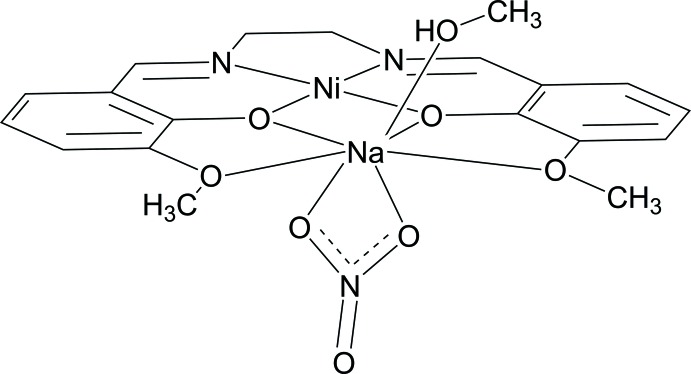


In the case of compartmental Schiff base ligands such as *e.g.* N(imine)_2_O(phenoxo)_2_O(meth­oxy/eth­oxy)_2_, the metal ion may be either retained in the plane of the O_4_ donor set or sandwiched between two sets of the Schiff base O atoms. The former case is usually characterized by a coordination number of eight from two O(phenoxo)_2_O(meth­oxy/eth­oxy)_2_ com­part­ments which belong to different mol­ecules. The latter features a coordination number of six from the O_4_ compartment of the Schiff base, and two other donors are provided by coordinating solvent mol­ecules and/or anions. The present paper is devoted to the synthesis and structural analysis of an Ni^2+^-containing complex [NaNi(*L*)(CH_3_OH)(NO_3_)], (I), in which the Na^+^ ion has a seven-coordination geometry and where H_2_ *L* is the compartmental Schiff base ligand 6,6′-dimeth­oxy-2,2′-(ethane-1,2-diyldi­imino­dimethyl­ene)diphenol.

## Structural commentary   

**Figure 1:**
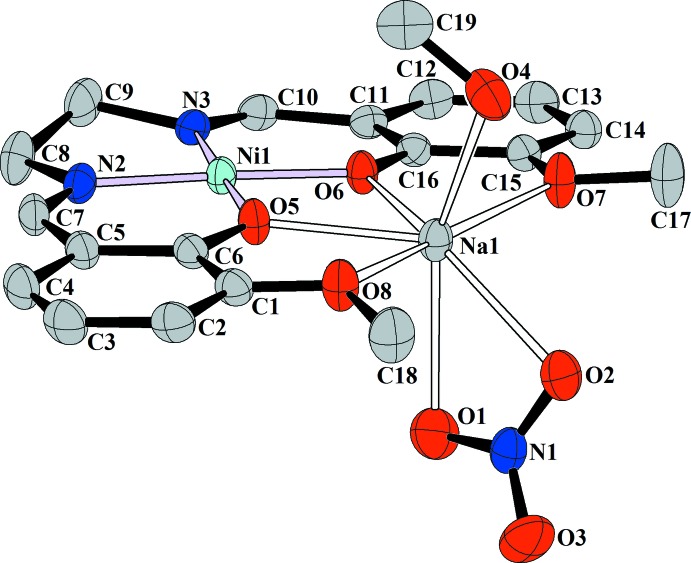
The mol­ecular structure of (I), showing 30% probability displacement ellipsoids and the atom-numbering scheme. H atoms have been omitted for clarity.

The mol­ecular structure of compound (I) with the atom numbering is shown in Fig. 1 ▶. Two phenolate O atoms provided by the Schiff base ligand create a double bridge between the Ni^2+^ and Na^+^ ions. The coordination environment of the Ni^2+^ ion is square-planar, formed by two imine N atoms and two phenolate O atoms. The Na^+^ ion has an unusual seven-coordinated geometry in which the ion sits in the plane of the Schiff base O atoms. Further significant inter­actions with two nitrate O atoms and one O atom from the coordin­ating methanol mol­ecule, which are located above and below the plane formed by *L*, complete the coordination sphere. Values for the geometric parameters in (I) are in good agreement with those observed for complexes based on similar Schiff base ligands (Allen *et al.*, 1987 ▶; Cunningham *et al.*, 2000 ▶; Wang & Shen, 2009 ▶; Xiao, 2009 ▶). The two phenoxo and two meth­oxy O atoms of the O(phenoxo)_2_O(eth­oxy)_2_ moiety adopt a planar geometry as evidenced by the small mean deviation of the O atoms (<0.02 Å), from the O5/O6/O7/O8 least-squares plane. The deviations of the Na^+^ and Ni^2+^ ions from the O5/O6/O7/O8 plane [0.166 (1) and 0.008 (2) Å, respectively] indicate that Na and Ni are well incorporated in the O(phenoxo)_2_O(eth­oxy)_2_ moiety.

## Supra­molecular features   

**Figure 2:**
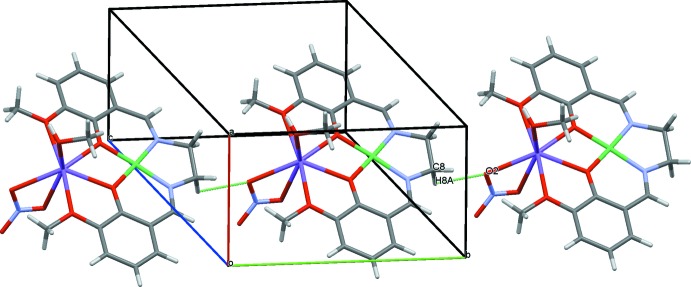
The mol­ecular packing for (I), viewed along the *b* axis. C—H⋯O inter­actions are shown as dashed lines.

**Figure 3:**
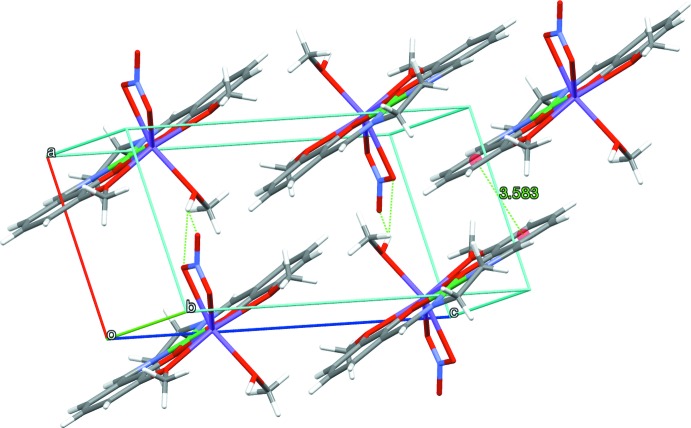
O—H⋯O and π–π contacts for (I), shown as dashed lines, with ring centroids shown as coloured spheres.

In the crystal structure, the mol­ecules of the title compound form chains along the *b*-axis *via* weak C—H⋯O hydrogen-bond inter­actions (Fig. 2 ▶, Table 1 ▶). The C atom of the ethyl­ene moiety acts as a donor and one O atom of the nitrate anion of the neighboring mol­ecule acts as an acceptor. These chains are further assembled into sheets by a bifurcated O—H⋯O hydrogen bond (Steiner, 2002 ▶), which involves the coordin­ating methanol mol­ecule and nitrate units (Fig. 3 ▶, Table 1 ▶) and through π–π stacking inter­actions, which exist between phenyl rings of neighbouring mol­ecules, with a separation of 3.5845 (11) Å between the centroids formed by the C atoms of the rings [symmetry code: (iii) −*x* + 1, −*y*, −*z*]. For the O—H⋯O hydrogen bond, the O atom of the methanol mol­ecule acts as a donor and the O atoms of the nitrate anion of the neighbouring mol­ecule act as the acceptors.

## Synthesis and crystallization   

A mixture of 6,6′-dimeth­oxy-2,2′-(ethane-1,2-diyldi­imino­dimethyl­ene)diphenol (1 mmol) and nickel nitrate (1 mmol) in methanol (15 ml) was stirred for 30 min at room temperature. Then, sodium nitrate (1mmol) was added, and the mixture was stirred for another 30 min and filtered. The resulting clear orange filtrate was left at ambient temperature for crystallization in air. The red–orange block-shaped crystals were collected by filtration after 6 d, washed with chilled iso­propanol and dried on filter paper (yield 0.28 g, 56%).

## Refinement   

H atoms were placed in geometrically idealized positions and constrained to ride on their parent atoms, with C—H distances of 0.95 (aromatic) or 0.99 Å (methyl­ene), with *U*_iso_(H) = 1.2*U*_eq_(C), C—H = 0.98 Å for methyl H atoms, with *U*_iso_(H) = 1.5*U*_eq_(C), and O—H = 0.82 Å for the hy­droxy group of methanol, with *U*_iso_(H) = 1.5*U*_eq_(O). Crystal data, data collection and structure refinement details are summarized in Table 2 ▶.

## Supplementary Material

Crystal structure: contains datablock(s) I. DOI: 10.1107/S160053681402159X/zl2599sup1.cif

Structure factors: contains datablock(s) I. DOI: 10.1107/S160053681402159X/zl2599Isup2.hkl

CCDC reference: 1026857

Additional supporting information:  crystallographic information; 3D view; checkCIF report

## Figures and Tables

**Table 1:** Hydrogen-bond geometry (, )

*D*H*A*	*D*H	H*A*	*D* *A*	*D*H*A*
O4H4*O*O1^i^	0.82	2.24	2.991(2)	154
O4H4*O*O3^i^	0.82	2.49	3.181(2)	143
C8H8*B*O2^ii^	0.97	2.65	3.152(2)	112

Symmetry codes: (i) 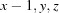; (ii) 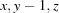.

**Table 2:** Experimental details

Crystal data	
Chemical formula	[NaNi(C_18_H_18_N_2_O_4_)(NO_3_)(CH_4_O)]
*M*_r_	502.09
Crystal system, space group	Triclinic, *P* 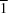
Temperature (K)	293
*a*, *b*, *c* ()	7.207(1), 11.047(1), 13.619(1)
, , ()	95.30(1), 99.81(1), 99.05(1)
*V* (^3^)	1047.2(2)
*Z*	2
Radiation type	Mo *K*
(mm^1^)	1.00
Crystal size (mm)	0.4 0.2 0.2

Data collection	
Diffractometer	Nonius KappaCCD
Absorption correction	Multi-scan (*SADABS*; Sheldrick, 2003 ▶)
*T*_min_, *T*_max_	0.690, 0.825
No. of measured, independent and observed [*I* > 2(*I*)] reflections	12718, 6501, 4324
*R*_int_	0.020
(sin /)_max_ (^1^)	0.744

Refinement	
*R*[*F* ^2^ > 2(*F* ^2^)], *wR*(*F* ^2^), *S*	0.035, 0.081, 0.90
No. of reflections	6501
No. of parameters	292
H-atom treatment	H-atom parameters constrained
_max_, _min_ (e ^3^)	0.50, 0.32

Computer programs: *COLLECT* (Nonius, 1999 ▶), *DENZO*/*SCALEPACK* (Otwinowski Minor, 1997 ▶), *SHELXS97* and *SHELXL2014* (Sheldrick, 2008 ▶), *ORTEP-3 for Windows* and *WinGX* (Farrugia, 2012 ▶).

## supplementary crystallographic information

### Crystal data

**Table:**

[NaNi(C_18_H_18_N_2_O_4_)(NO_3_)(CH_4_O)]	*Z* = 2
*M**_r_* = 502.09	*F*(000) = 520
Triclinic, *P*1	*D*_x_ = 1.592 Mg m^−^^3^
*a* = 7.207 (1) Å	Mo *K*α radiation, λ = 0.71073 Å
*b* = 11.047 (1) Å	Cell parameters from 12718 reflections
*c* = 13.619 (1) Å	θ = 2.9–31.9°
α = 95.30 (1)°	µ = 1.00 mm^−^^1^
β = 99.81 (1)°	*T* = 293 K
γ = 99.05 (1)°	Block, white
*V* = 1047.2 (2) Å^3^	0.4 × 0.2 × 0.2 mm

### Data collection

**Table:**

Nonius KappaCCD diffractometer	4324 reflections with *I* > 2σ(*I*)
Radiation source: sealed X-ray tube	*R*_int_ = 0.020
φ scans and ω scans with κ offset	θ_max_ = 31.9°, θ_min_ = 2.9°
Absorption correction: multi-scan (*SADABS*; Sheldrick, 2003)	*h* = −8→10
*T*_min_ = 0.690, *T*_max_ = 0.825	*k* = −15→15
12718 measured reflections	*l* = −19→20
6501 independent reflections	

### Refinement

**Table:**

Refinement on *F*^2^	0 restraints
Least-squares matrix: full	Hydrogen site location: mixed
*R*[*F*^2^ > 2σ(*F*^2^)] = 0.035	H-atom parameters constrained
*wR*(*F*^2^) = 0.081	*w* = 1/[σ^2^(*F*_o_^2^) + (0.0431*P*)^2^] where *P* = (*F*_o_^2^ + 2*F*_c_^2^)/3
*S* = 0.90	(Δ/σ)_max_ < 0.001
6501 reflections	Δρ_max_ = 0.50 e Å^−^^3^
292 parameters	Δρ_min_ = −0.32 e Å^−^^3^

### Special details

**Table:**

Geometry. All e.s.d.'s (except the e.s.d. in the dihedral angle between two l.s. planes) are estimated using the full covariance matrix. The cell e.s.d.'s are taken into account individually in the estimation of e.s.d.'s in distances, angles and torsion angles; correlations between e.s.d.'s in cell parameters are only used when they are defined by crystal symmetry. An approximate (isotropic) treatment of cell e.s.d.'s is used for estimating e.s.d.'s involving l.s. planes.
Refinement. Refinement of *F*^2^ against ALL reflections. The weighted *R*-factor *wR* and goodness of fit *S* are based on *F*^2^, conventional *R*-factors *R* are based on *F*, with *F* set to zero for negative *F*^2^. The threshold expression of *F*^2^ > σ(*F*^2^) is used only for calculating *R*-factors(gt) *etc*. and is not relevant to the choice of reflections for refinement. *R*-factors based on *F*^2^ are statistically about twice as large as those based on *F*, and *R*- factors based on ALL data will be even larger.

### Fractional atomic coordinates and isotropic or equivalent isotropic displacement parameters (Å^2^)

**Table:**

	*x*	*y*	*z*	*U*_iso_*/*U*_eq_	
N1	1.3085 (3)	0.47914 (14)	0.20473 (11)	0.0500 (4)	
N2	1.0634 (2)	−0.10231 (12)	0.31510 (10)	0.0414 (3)	
N3	0.87587 (19)	−0.13606 (11)	0.13663 (10)	0.0384 (3)	
O1	1.3115 (2)	0.36716 (13)	0.19219 (14)	0.0823 (5)	
O2	1.1514 (2)	0.51204 (13)	0.20629 (12)	0.0692 (4)	
O3	1.4575 (2)	0.55470 (16)	0.21536 (12)	0.0840 (5)	
O4	0.7094 (2)	0.36010 (15)	0.29641 (10)	0.0708 (4)	
H4O	0.6210	0.3784	0.2582	0.106*	
O5	1.04924 (18)	0.14171 (10)	0.31975 (8)	0.0439 (3)	
O6	0.87634 (17)	0.10986 (10)	0.14652 (8)	0.0401 (3)	
O7	0.7790 (2)	0.29628 (11)	0.06339 (9)	0.0531 (3)	
O8	1.1398 (2)	0.36213 (11)	0.41444 (9)	0.0539 (3)	
C1	1.2043 (2)	0.27108 (16)	0.46557 (12)	0.0417 (4)	
C2	1.3145 (3)	0.28976 (19)	0.56084 (13)	0.0504 (4)	
H2	1.3492	0.3688	0.5958	0.060*	
C3	1.3734 (3)	0.1895 (2)	0.60439 (14)	0.0592 (5)	
H3	1.4487	0.2022	0.6683	0.071*	
C4	1.3227 (3)	0.0743 (2)	0.55493 (13)	0.0554 (5)	
H4	1.3625	0.0085	0.5856	0.067*	
C5	1.2091 (2)	0.05142 (16)	0.45629 (12)	0.0419 (4)	
C6	1.1504 (2)	0.15157 (15)	0.41092 (11)	0.0380 (4)	
C7	1.1604 (3)	−0.07053 (17)	0.40535 (13)	0.0457 (4)	
H7	1.2019	−0.1330	0.4399	0.055*	
C8	1.0406 (3)	−0.23159 (17)	0.27149 (14)	0.0585 (5)	
H8A	1.0011	−0.2860	0.3189	0.070*	
H8B	1.1616	−0.2488	0.2568	0.070*	
C9	0.8924 (3)	−0.25411 (15)	0.17663 (15)	0.0578 (5)	
H9A	0.9287	−0.3105	0.1274	0.069*	
H9B	0.7699	−0.2914	0.1905	0.069*	
C10	0.7997 (2)	−0.13762 (15)	0.04360 (13)	0.0411 (4)	
H10	0.7711	−0.2136	0.0037	0.049*	
C11	0.7552 (2)	−0.03232 (15)	−0.00347 (11)	0.0366 (3)	
C12	0.6631 (3)	−0.04768 (17)	−0.10544 (12)	0.0455 (4)	
H12	0.6384	−0.1255	−0.1424	0.055*	
C13	0.6106 (3)	0.04949 (18)	−0.14969 (12)	0.0489 (4)	
H13	0.5500	0.0374	−0.2167	0.059*	
C14	0.6459 (2)	0.16813 (16)	−0.09638 (12)	0.0419 (4)	
H14	0.6087	0.2342	−0.1276	0.050*	
C15	0.7366 (2)	0.18574 (15)	0.00292 (11)	0.0372 (4)	
C16	0.7923 (2)	0.08597 (14)	0.05155 (11)	0.0340 (3)	
C17	0.7114 (4)	0.39946 (18)	0.02595 (15)	0.0719 (7)	
H17A	0.7727	0.4218	−0.0287	0.108*	
H17B	0.7399	0.4676	0.0784	0.108*	
H17C	0.5755	0.3791	0.0027	0.108*	
C18	1.2117 (4)	0.48674 (18)	0.45544 (16)	0.0714 (7)	
H18A	1.1842	0.5002	0.5217	0.107*	
H18B	1.1519	0.5405	0.4136	0.107*	
H18C	1.3475	0.5039	0.4587	0.107*	
C19	0.6357 (4)	0.2606 (2)	0.34481 (17)	0.0756 (7)	
H19A	0.6104	0.1856	0.2994	0.113*	
H19B	0.5192	0.2755	0.3650	0.113*	
H19C	0.7275	0.2531	0.4029	0.113*	
Ni1	0.96567 (3)	0.00143 (2)	0.22924 (2)	0.03524 (7)	
Na1	0.97774 (10)	0.30835 (6)	0.23162 (5)	0.04288 (16)	

### Atomic displacement parameters (Å^2^)

**Table:**

	*U*^11^	*U*^22^	*U*^33^	*U*^12^	*U*^13^	*U*^23^
N1	0.0603 (11)	0.0439 (9)	0.0431 (8)	0.0079 (8)	0.0013 (7)	0.0085 (7)
N2	0.0517 (9)	0.0324 (7)	0.0464 (8)	0.0153 (6)	0.0169 (7)	0.0103 (6)
N3	0.0379 (8)	0.0283 (7)	0.0493 (8)	0.0060 (5)	0.0091 (6)	0.0051 (6)
O1	0.0755 (12)	0.0449 (9)	0.1300 (14)	0.0208 (8)	0.0217 (10)	0.0081 (9)
O2	0.0728 (11)	0.0523 (9)	0.0915 (11)	0.0276 (8)	0.0203 (8)	0.0175 (7)
O3	0.0702 (12)	0.0779 (11)	0.0851 (11)	−0.0239 (9)	−0.0063 (8)	0.0156 (9)
O4	0.0659 (10)	0.0912 (11)	0.0610 (9)	0.0337 (9)	0.0065 (7)	0.0143 (8)
O5	0.0570 (8)	0.0342 (6)	0.0367 (6)	0.0132 (5)	−0.0063 (5)	0.0041 (5)
O6	0.0512 (7)	0.0295 (6)	0.0350 (6)	0.0094 (5)	−0.0055 (5)	0.0019 (4)
O7	0.0809 (10)	0.0335 (6)	0.0400 (6)	0.0180 (6)	−0.0087 (6)	0.0025 (5)
O8	0.0715 (9)	0.0379 (7)	0.0434 (7)	0.0095 (6)	−0.0106 (6)	−0.0001 (5)
C1	0.0384 (10)	0.0475 (10)	0.0373 (8)	0.0068 (7)	0.0027 (7)	0.0043 (7)
C2	0.0466 (11)	0.0605 (12)	0.0384 (9)	0.0031 (9)	0.0000 (8)	0.0025 (8)
C3	0.0523 (13)	0.0792 (15)	0.0418 (10)	0.0118 (10)	−0.0052 (8)	0.0112 (10)
C4	0.0549 (13)	0.0717 (14)	0.0440 (10)	0.0226 (10)	0.0022 (8)	0.0227 (9)
C5	0.0403 (10)	0.0495 (10)	0.0397 (9)	0.0145 (8)	0.0076 (7)	0.0131 (7)
C6	0.0339 (9)	0.0467 (10)	0.0344 (8)	0.0092 (7)	0.0049 (6)	0.0089 (7)
C7	0.0502 (11)	0.0501 (11)	0.0467 (10)	0.0230 (8)	0.0145 (8)	0.0231 (8)
C8	0.0886 (16)	0.0380 (10)	0.0563 (11)	0.0250 (10)	0.0168 (10)	0.0146 (8)
C9	0.0685 (14)	0.0264 (9)	0.0742 (13)	0.0049 (8)	0.0038 (10)	0.0070 (8)
C10	0.0410 (10)	0.0290 (8)	0.0507 (10)	0.0018 (7)	0.0107 (7)	−0.0052 (7)
C11	0.0338 (9)	0.0360 (8)	0.0386 (8)	0.0031 (6)	0.0076 (6)	0.0006 (6)
C12	0.0492 (11)	0.0443 (10)	0.0379 (9)	0.0016 (8)	0.0075 (7)	−0.0079 (7)
C13	0.0497 (11)	0.0615 (12)	0.0309 (8)	0.0046 (9)	0.0022 (7)	0.0003 (8)
C14	0.0421 (10)	0.0480 (10)	0.0357 (8)	0.0098 (7)	0.0044 (7)	0.0083 (7)
C15	0.0378 (9)	0.0377 (9)	0.0352 (8)	0.0079 (7)	0.0036 (6)	0.0043 (6)
C16	0.0326 (9)	0.0327 (8)	0.0346 (8)	0.0039 (6)	0.0041 (6)	0.0009 (6)
C17	0.111 (2)	0.0414 (11)	0.0596 (12)	0.0280 (11)	−0.0082 (12)	0.0091 (9)
C18	0.0865 (18)	0.0455 (12)	0.0671 (13)	0.0025 (11)	−0.0136 (12)	−0.0035 (10)
C19	0.0713 (17)	0.0801 (17)	0.0719 (15)	0.0061 (13)	0.0154 (12)	0.0002 (13)
Ni1	0.03972 (13)	0.02768 (11)	0.03891 (12)	0.00921 (8)	0.00475 (8)	0.00648 (8)
Na1	0.0529 (4)	0.0312 (3)	0.0421 (3)	0.0085 (3)	0.0013 (3)	0.0039 (3)

### Geometric parameters (Å, º)

**Table:**

N1—O3	1.230 (2)	C4—C5	1.427 (2)
N1—O1	1.2372 (19)	C4—H4	0.9300
N1—O2	1.246 (2)	C5—C6	1.403 (2)
N1—Na1	2.8961 (19)	C5—C7	1.420 (2)
N2—C7	1.293 (2)	C7—H7	0.9300
N2—C8	1.468 (2)	C8—C9	1.503 (3)
N2—Ni1	1.8433 (13)	C8—H8A	0.9700
N3—C10	1.290 (2)	C8—H8B	0.9700
N3—C9	1.473 (2)	C9—H9A	0.9700
N3—Ni1	1.8371 (13)	C9—H9B	0.9700
O1—Na1	2.5512 (19)	C10—C11	1.432 (2)
O2—Na1	2.4806 (16)	C10—H10	0.9300
O4—C19	1.412 (3)	C11—C16	1.408 (2)
O4—Na1	2.3837 (17)	C11—C12	1.416 (2)
O4—H4O	0.8151	C12—C13	1.352 (3)
O5—C6	1.3139 (18)	C12—H12	0.9300
O5—Ni1	1.8396 (11)	C13—C14	1.402 (2)
O5—Na1	2.3644 (12)	C13—H13	0.9300
O6—C16	1.3148 (17)	C14—C15	1.380 (2)
O6—Ni1	1.8339 (10)	C14—H14	0.9300
O6—Na1	2.3288 (12)	C15—C16	1.413 (2)
O7—C15	1.3689 (19)	C17—H17A	0.9600
O7—C17	1.412 (2)	C17—H17B	0.9600
O7—Na1	2.4666 (13)	C17—H17C	0.9600
O8—C1	1.371 (2)	C18—H18A	0.9600
O8—C18	1.418 (2)	C18—H18B	0.9600
O8—Na1	2.5364 (13)	C18—H18C	0.9600
C1—C2	1.380 (2)	C19—Na1	3.125 (3)
C1—C6	1.416 (2)	C19—H19A	0.9600
C2—C3	1.394 (3)	C19—H19B	0.9600
C2—H2	0.9300	C19—H19C	0.9600
C3—C4	1.349 (3)	Ni1—Na1	3.3749 (7)
C3—H3	0.9300		
			
O3—N1—O1	120.31 (19)	O6—C16—C11	123.80 (14)
O3—N1—O2	121.67 (18)	O6—C16—C15	117.42 (13)
O1—N1—O2	118.02 (17)	C11—C16—C15	118.78 (13)
O3—N1—Na1	166.28 (12)	O7—C17—H17A	109.5
O1—N1—Na1	61.59 (11)	O7—C17—H17B	109.5
O2—N1—Na1	58.35 (10)	H17A—C17—H17B	109.5
C7—N2—C8	118.06 (14)	O7—C17—H17C	109.5
C7—N2—Ni1	126.55 (12)	H17A—C17—H17C	109.5
C8—N2—Ni1	115.16 (11)	H17B—C17—H17C	109.5
C10—N3—C9	118.99 (14)	O8—C18—H18A	109.5
C10—N3—Ni1	126.49 (11)	O8—C18—H18B	109.5
C9—N3—Ni1	114.51 (11)	H18A—C18—H18B	109.5
N1—O1—Na1	93.16 (12)	O8—C18—H18C	109.5
N1—O2—Na1	96.34 (11)	H18A—C18—H18C	109.5
C19—O4—Na1	108.10 (13)	H18B—C18—H18C	109.5
C19—O4—H4O	108.2	O4—C19—Na1	46.47 (10)
Na1—O4—H4O	119.0	O4—C19—H19A	109.5
C6—O5—Ni1	127.75 (10)	Na1—C19—H19A	79.9
C6—O5—Na1	125.60 (10)	O4—C19—H19B	109.5
Ni1—O5—Na1	106.12 (5)	Na1—C19—H19B	155.4
C16—O6—Ni1	127.73 (10)	H19A—C19—H19B	109.5
C16—O6—Na1	123.96 (9)	O4—C19—H19C	109.5
Ni1—O6—Na1	107.75 (5)	Na1—C19—H19C	87.4
C15—O7—C17	118.69 (13)	H19A—C19—H19C	109.5
C15—O7—Na1	118.60 (9)	H19B—C19—H19C	109.5
C17—O7—Na1	122.69 (10)	O6—Ni1—N3	95.02 (5)
C1—O8—C18	118.12 (13)	O6—Ni1—O5	83.21 (5)
C1—O8—Na1	118.85 (9)	N3—Ni1—O5	178.04 (5)
C18—O8—Na1	120.66 (11)	O6—Ni1—N2	177.63 (6)
O8—C1—C2	125.03 (16)	N3—Ni1—N2	87.08 (6)
O8—C1—C6	113.83 (13)	O5—Ni1—N2	94.71 (6)
C2—C1—C6	121.13 (16)	O6—Ni1—Na1	41.09 (3)
C1—C2—C3	119.59 (18)	N3—Ni1—Na1	135.99 (4)
C1—C2—H2	120.2	O5—Ni1—Na1	42.30 (3)
C3—C2—H2	120.2	N2—Ni1—Na1	136.74 (5)
C4—C3—C2	120.82 (16)	O6—Na1—O5	62.62 (4)
C4—C3—H3	119.6	O6—Na1—O4	105.64 (6)
C2—C3—H3	119.6	O5—Na1—O4	102.21 (5)
C3—C4—C5	121.15 (18)	O6—Na1—O7	64.88 (4)
C3—C4—H4	119.4	O5—Na1—O7	127.21 (5)
C5—C4—H4	119.4	O4—Na1—O7	86.56 (5)
C6—C5—C7	121.30 (15)	O6—Na1—O2	139.36 (6)
C6—C5—C4	118.75 (17)	O5—Na1—O2	136.66 (5)
C7—C5—C4	119.95 (16)	O4—Na1—O2	103.03 (6)
O5—C6—C5	123.93 (15)	O7—Na1—O2	88.99 (5)
O5—C6—C1	117.52 (14)	O6—Na1—O8	125.89 (5)
C5—C6—C1	118.55 (14)	O5—Na1—O8	63.39 (4)
N2—C7—C5	125.72 (15)	O4—Na1—O8	82.22 (5)
N2—C7—H7	117.1	O7—Na1—O8	166.29 (5)
C5—C7—H7	117.1	O2—Na1—O8	85.91 (5)
N2—C8—C9	109.13 (14)	O6—Na1—O1	102.51 (5)
N2—C8—H8A	109.9	O5—Na1—O1	95.38 (5)
C9—C8—H8A	109.9	O4—Na1—O1	151.34 (6)
N2—C8—H8B	109.9	O7—Na1—O1	100.68 (6)
C9—C8—H8B	109.9	O2—Na1—O1	50.02 (5)
H8A—C8—H8B	108.3	O8—Na1—O1	85.75 (6)
N3—C9—C8	109.49 (14)	O6—Na1—N1	124.94 (5)
N3—C9—H9A	109.8	O5—Na1—N1	114.82 (5)
C8—C9—H9A	109.8	O4—Na1—N1	126.53 (6)
N3—C9—H9B	109.8	O7—Na1—N1	98.99 (5)
C8—C9—H9B	109.8	O2—Na1—N1	25.31 (4)
H9A—C9—H9B	108.2	O8—Na1—N1	81.61 (5)
N3—C10—C11	125.54 (14)	O1—Na1—N1	25.25 (4)
N3—C10—H10	117.2	O6—Na1—C19	88.26 (6)
C11—C10—H10	117.2	O5—Na1—C19	77.74 (6)
C16—C11—C12	119.08 (15)	O4—Na1—C19	25.43 (6)
C16—C11—C10	120.98 (14)	O7—Na1—C19	95.80 (6)
C12—C11—C10	119.84 (14)	O2—Na1—C19	126.74 (6)
C13—C12—C11	120.74 (15)	O8—Na1—C19	77.25 (6)
C13—C12—H12	119.6	O1—Na1—C19	162.98 (6)
C11—C12—H12	119.6	N1—Na1—C19	146.80 (6)
C12—C13—C14	121.20 (15)	O6—Na1—Ni1	31.17 (3)
C12—C13—H13	119.4	O5—Na1—Ni1	31.58 (3)
C14—C13—H13	119.4	O4—Na1—Ni1	108.42 (5)
C15—C14—C13	119.21 (16)	O7—Na1—Ni1	96.03 (3)
C15—C14—H14	120.4	O2—Na1—Ni1	148.38 (4)
C13—C14—H14	120.4	O8—Na1—Ni1	94.97 (3)
O7—C15—C14	125.11 (15)	O1—Na1—Ni1	98.44 (4)
O7—C15—C16	113.90 (12)	N1—Na1—Ni1	123.52 (4)
C14—C15—C16	120.99 (15)	C19—Na1—Ni1	83.90 (5)
			
O3—N1—O1—Na1	164.40 (14)	C12—C13—C14—C15	0.3 (3)
O2—N1—O1—Na1	−15.51 (17)	C17—O7—C15—C14	6.0 (3)
O3—N1—O2—Na1	−163.87 (15)	Na1—O7—C15—C14	−172.37 (14)
O1—N1—O2—Na1	16.04 (18)	C17—O7—C15—C16	−173.20 (18)
C18—O8—C1—C2	9.2 (3)	Na1—O7—C15—C16	8.43 (19)
Na1—O8—C1—C2	171.84 (14)	C13—C14—C15—O7	−179.79 (16)
C18—O8—C1—C6	−169.82 (18)	C13—C14—C15—C16	−0.6 (3)
Na1—O8—C1—C6	−7.19 (19)	Ni1—O6—C16—C11	0.5 (2)
O8—C1—C2—C3	−179.16 (17)	Na1—O6—C16—C11	170.80 (12)
C6—C1—C2—C3	−0.2 (3)	Ni1—O6—C16—C15	−179.59 (11)
C1—C2—C3—C4	−0.6 (3)	Na1—O6—C16—C15	−9.3 (2)
C2—C3—C4—C5	0.6 (3)	C12—C11—C16—O6	179.76 (15)
C3—C4—C5—C6	0.1 (3)	C10—C11—C16—O6	3.2 (3)
C3—C4—C5—C7	178.98 (19)	C12—C11—C16—C15	−0.2 (2)
Ni1—O5—C6—C5	−1.7 (2)	C10—C11—C16—C15	−176.70 (15)
Na1—O5—C6—C5	−172.10 (12)	O7—C15—C16—O6	−0.1 (2)
Ni1—O5—C6—C1	177.62 (12)	C14—C15—C16—O6	−179.34 (15)
Na1—O5—C6—C1	7.2 (2)	O7—C15—C16—C11	179.82 (15)
C7—C5—C6—O5	−0.5 (3)	C14—C15—C16—C11	0.6 (2)
C4—C5—C6—O5	178.43 (16)	C16—O6—Ni1—N3	−4.68 (14)
C7—C5—C6—C1	−179.72 (16)	Na1—O6—Ni1—N3	−176.25 (6)
C4—C5—C6—C1	−0.8 (3)	C16—O6—Ni1—O5	176.15 (14)
O8—C1—C6—O5	0.7 (2)	Na1—O6—Ni1—O5	4.58 (6)
C2—C1—C6—O5	−178.41 (16)	C16—O6—Ni1—Na1	171.57 (17)
O8—C1—C6—C5	179.98 (15)	C10—N3—Ni1—O6	7.18 (15)
C2—C1—C6—C5	0.9 (3)	C9—N3—Ni1—O6	−171.60 (13)
C8—N2—C7—C5	174.90 (17)	C10—N3—Ni1—N2	−171.71 (15)
Ni1—N2—C7—C5	0.6 (3)	C9—N3—Ni1—N2	9.50 (13)
C6—C5—C7—N2	1.0 (3)	C10—N3—Ni1—Na1	3.64 (18)
C4—C5—C7—N2	−177.89 (17)	C9—N3—Ni1—Na1	−175.15 (10)
C7—N2—C8—C9	168.53 (17)	C6—O5—Ni1—O6	−176.40 (15)
Ni1—N2—C8—C9	−16.6 (2)	Na1—O5—Ni1—O6	−4.48 (6)
C10—N3—C9—C8	160.57 (16)	C6—O5—Ni1—N2	2.46 (15)
Ni1—N3—C9—C8	−20.5 (2)	Na1—O5—Ni1—N2	174.39 (6)
N2—C8—C9—N3	22.7 (2)	C6—O5—Ni1—Na1	−171.92 (17)
C9—N3—C10—C11	173.00 (16)	C7—N2—Ni1—N3	178.85 (16)
Ni1—N3—C10—C11	−5.7 (3)	C8—N2—Ni1—N3	4.44 (14)
N3—C10—C11—C16	−0.5 (3)	C7—N2—Ni1—O5	−1.95 (16)
N3—C10—C11—C12	−177.00 (17)	C8—N2—Ni1—O5	−176.35 (13)
C16—C11—C12—C13	−0.2 (3)	C7—N2—Ni1—Na1	3.57 (19)
C10—C11—C12—C13	176.39 (16)	C8—N2—Ni1—Na1	−170.84 (11)
C11—C12—C13—C14	0.1 (3)		

### Hydrogen-bond geometry (Å, º)

**Table:**

*D*—H···*A*	*D*—H	H···*A*	*D*···*A*	*D*—H···*A*
O4—H4*O*···O1^i^	0.82	2.24	2.991 (2)	154
O4—H4*O*···O3^i^	0.82	2.49	3.181 (2)	143
C8—H8*B*···O2^ii^	0.97	2.65	3.152 (2)	112

Symmetry codes: (i) *x*−1, *y*, *z*; (ii) *x*, *y*−1, *z*.
